# Effect of Dance Movement Therapy on Cancer-Related Fatigue in Breast Cancer Patients Undergoing Radiation Therapy: A Pre-post Intervention Study

**DOI:** 10.7759/cureus.21040

**Published:** 2022-01-09

**Authors:** Vishnu Vardhan, Chanan Goyal, Jaimini Chaudhari, Vandana Jain, Chaitanya A Kulkarni, Moli Jain

**Affiliations:** 1 Cardiorespiratory Physiotherapy, Datta Meghe Institute of Medical Sciences, Wardha, IND; 2 Paediatric Physiotherapy, Datta Meghe Institute of Medical Sciences, Wardha, IND; 3 Paediatric Physiotherapy, Government Physiotherapy College, Raipur, IND; 4 Physiotherapy, Pravara Institute of Medical Sciences, Ahmednagar, IND; 5 Radiation Oncology, Pravara Institute of Medical Sciences, Ahmednagar, IND; 6 Community Physiotherapy, Datta Meghe Institute of Medical Sciences, Wardha, IND

**Keywords:** cancer-related fatigue, physiotherapy, radiation therapy, creative movement therapy, dance movement therapy, brief fatigue inventory, breast cancer

## Abstract

Introduction

Dance movement therapy (DMT) is a movement-based psychosocial intervention that incorporates the therapeutic components of dance movements and group psychotherapy. DMT, also known as creative movement therapy (CMT) is a psychotherapy used as a complementary therapy in cancer care. It helps in enhancing mood, emotions, self-expression and helps to rebuild self-confidence. Besides, it allows the patients to recognise their own strengths and weaknesses as well as helps to improve physical capabilities.

Methods

By simple random sampling method, 30 breast cancer patients were recruited at Pravara Rural Hospital, Loni, Maharashtra, India. The participants were in the age range of 30-60 years based on the inclusion and exclusion criteria. Pre-intervention scores of cancer-related fatigue (CRF) were taken using the Brief Fatigue Inventory (BFI) scale and intervention was given for 45 minutes each day for 5 days a week, over a span of 2 weeks. Thereafter, post-intervention assessment was done and the scores were noted. Pre-intervention and post-intervention scores were compared using paired t-test.

Results

The mean and standard deviation (SD) of pre- and post-BFI scores derived by using paired t-test was 73.76 (8.6) and 69.33 (9.8), respectively, with a p-value of < 0.001, which is highly significant.

Conclusion

The results of the present study revealed that DMT seems to be effective in reducing some amount of CRF in breast cancer patients undergoing radiation therapy. Besides, it turned out to be an engaging, entertaining and cost-effective approach. The investigation showed that DMT appears to be beneficial in reducing the side effects of radiation therapy such as pain, stress, anxiety and fear, giving a psychotherapeutic relief but did not completely remove the persistent fatigue experienced by the breast cancer patients. Thus, further investigation with long-term follow-up is recommended.

## Introduction

Fatigue is characterised as a subjective sense of tiredness or inability to do certain tasks due to extreme weakness, which is a common and distressing symptom usually associated with cancer or cancer treatment and is termed as cancer-related fatigue (CRF). CRF differs from the fatigue experienced by a healthy individual because CRF is non-transient and is less likely to be relieved by rest as cancer patients experience constant fatigue [1]. CRF can be defined as “a painful, obstinate, subjective sense of physical, emotional and/or cognitive weakness or exhaustion related to cancer treatment that is not proportional to recent physical activity and interferes with usual functioning” [2]. Cancer patients reported having CRF from the time of diagnosis, throughout the treatment and even months or years after the completion of treatment [3]. CRF can affect the patient on multiple levels of psychological and physical functioning and usually causes a noticeable decrease in the patient’s quality of life [4].

Adjuvant radiation therapy (RT) is used to decrease the risk of relapse in women with breast cancer. Patients undertaking RT often experience multiple symptoms including pain, fatigue, stress, and sleep disturbance, with reported occurrence rates of over 60% near the end of RT. A large volume of research has found the usefulness of non-pharmacological interventions to alleviate treatment-related symptoms in cancer patients, use of physical movements along with the ongoing RT to relieve pain and to reduce CRF [5].

Dance movement therapy (DMT) is a movement-based psychosocial intervention that integrates the therapeutic components of dance movement and group psychotherapy. It empowers the patients to enhance self-expression, accept and reconnect with their own bodies, cope with feelings of depression and fear, rebuild the shattered self-confidence, and strengthen personal resources. The group approach permits them to share their emotions and concerns, and also coping strategies with others [6,7]. DMT can be defined as the therapeutic use of movements that further enhances emotional, cognitive, and physical and the social integration of the individual [8].

Application of DMT in group therapy helps to reduce the CRF in breast cancer patients [9]. Previous research showed that a brief DMT intervention over the course of RT would benefit the patients by buffering their treatment-related symptoms while the symptoms would worsen in women who did not receive DMT [10,11]. Studies revealed physical activity along with adjuvant therapies (RT) improves the cancer patients’ overall condition but there is a gap in understanding the reduction of CRF with the help of low-impact physical activity like DMT. The effects of DMT on patients receiving active cancer treatment are yet to be studied. More studies are needed to elucidate the effects of DMT on physical and psychological outcomes in cancer patients [12]. Active participation and movement-based physiotherapy have been observed to be beneficial in a variety of other common and rare disorders [13-15].

## Materials and methods

Prior to the commencement, the study was approved by the Institutional Review Board known as Institutional Ethical Committee of Dr. A.P.J. Abdul Kalam College of Physiotherapy at Pravara Institute of Medical Sciences with approval number BPT/INT/2019/05. Consent from the department of oncology and RT of Pravara Rural Hospital, Loni, India was also taken. Detailed information regarding the nature of study was given to the patients before participation. Written consent forms were taken from the patients and permissions were obtained. By simple randomised sampling, 30 breast cancer patients were recruited. Inclusion criteria included radical mastectomy patients undergoing RT for breast cancer between the age range of 30 and 60 years, having stage of cancer one to three with BFI score above 45 who were willing to participate in the study. Those patients fulfilling the inclusion criteria were requested to participate in the study. Subjects who had recent history of trauma, pregnant patients, patients with history of sleep apnoea or anaemia and the patients for whom exercise was contraindicated were excluded from the study. All the patients were given the Brief Fatigue Inventory (BFI) and were asked to fill the questionnaire, as a pre-intervention assessment and their scores were taken. BFI has been included in the Appendices section. Participants were informed that the use of the collected data is purely for the purpose of the study.

Outcome measure

BFI was the scale that was used to assess the severity of fatigue related to cancer, before and after the intervention. It is a valid and reliable scale to measure fatigue in patients with various chronic illnesses including cancer [16,17].

Procedure

The participants, in addition to the ongoing treatment, were given group therapy in the form of DMT. Each session lasted for 45 minutes and one such session was imparted on 5 days of a week for a duration of 2 weeks. Each session was divided into three sections namely warm-up phase, development phase and cool-down phase. Warm-up phase included introducing and greeting each other with welcoming gestures and breathing exercises for 5 minutes. Development phase is the most crucial because it challenges the patients to perform all the movements to the best of their abilities for around 30 minutes. As this study was conducted in a rural medical hospital, regional music was used allowing the patients to enjoy the rhythm. The patients were allowed to move and dance according to their comfort while following the steps as demonstrated by the physiotherapist. Cool-down phase was for 10 minutes which included sitting in circle holding hands and doing deep breathing exercises followed by meditation or sitting in silence. Last, everyone was allowed to share what they felt during the whole session.

Data collection

The outcome measure, BFI, is a nine-item, 11-point rating scale developed to assess subjective fatigue. The first three questions measure fatigue severity from 0 which indicates “no fatigue” to 10 which indicates “worst levels of fatigue.” The following six questions assess fatigue interference with daily activities including general activity, mood, walking ability, normal work (both inside and outside the home), relations with other people and enjoyment of life. Response options range from 0, indicating “does not interfere,” to 10 indicating, “completely interferes.” Higher scores on the BFI correspond to greater self-reported levels of fatigue. The participant has to indicate by circling how much fatigue they feel from 0 to 10 while doing some activity or during some situation. The participants were asked to fill this questionnaire before and after the 2 weeks’ sessions. The maximum score in BFI is 90. Table 1 shows the categorisation of fatigue according to the BFI score.

**Table 1:** Fatigue categories according to BFI scores BFI: Brief Fatigue Inventory

Fatigue Category	BFI Score
Mild	30-45
Moderate	45-75
Severe	75-90

## Results

**Figure 1:**
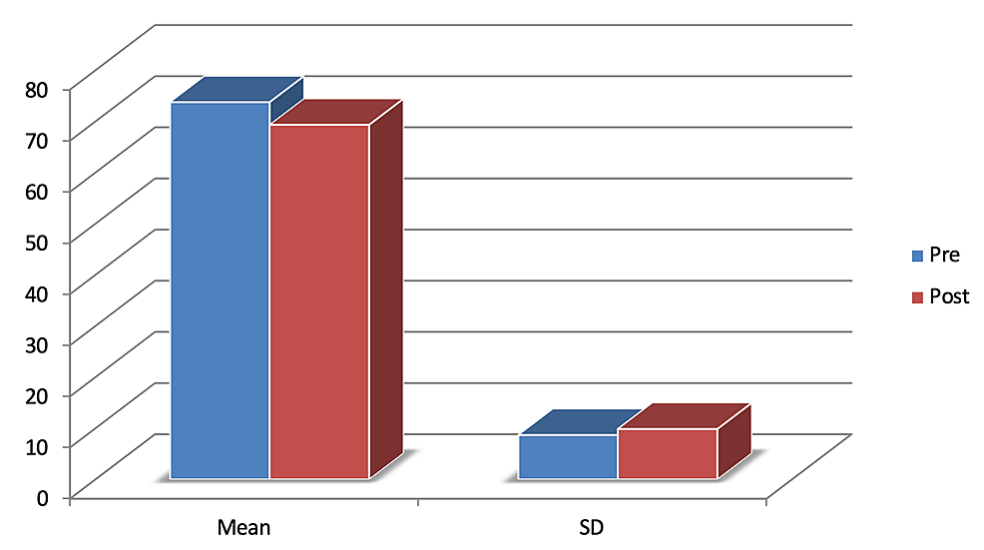
Graph showing pre- and post-intervention mean and standard deviation of BFI scores BFI: Brief Fatigue Inventory

A total of 30 participants were taken according to the selection criteria. Baseline characteristics (age and pre-interventional BFI scores) were taken and analysed. The mean age (±SD) was 45.66±8.83. The pre-intervention data collected showed the average (±SD) of 73.76±8.6 while the post-intervention data collected showed the average (±SD) of 69.33+9.8 as depicted in Figure 1.

The comparison of pre- and post-intervention BFI scores was found to be statistically significant using the paired t-test in which p-value was 0.001 (p>0.05) which is highly significant and t value was 2.62 as shown in Table 2.

**Table 2:** Pre- and post-intervention mean and standard deviation of BFI scores; t-value and p-value after performing paired t-test BFI: Brief Fatigue Intervention

	Pre	Post	Student’s paired t-test	p-value and significance
Mean	73.76 + 8.6	69.33 + 9.8	2.62	p = 0.001, highly significant
SD	8.67	9.85		

## Discussion

The effect of DMT has been studied in a variety of conditions including depression, dementia and cancer [18-21]. DMT has been investigated for cancer care, in both, children and adult population. Paediatric medical DMT is an approach that emphasises the expression of emotions through dance and movement [22]. Besides, different forms of dance are under consideration for therapeutic use for breast cancer patients with CRF [23,24]. Also, apart from group therapy, DMT has been utilised as an individualised therapy in a few studies [7,25]. Outcome measures and parameters used in various studies on use of DMT in patients with cancer included quality of life, anxiety, stress, pain, fatigue, vitality, body awareness, self-care and self-efficacy apart from similar others [7]. Fatigue is associated with cancer as well as with adjuvant cancer treatments like chemotherapy and radiotherapy but the pathogenesis of fatigue is largely unknown [26]. CRF is a frequently seen yet under-diagnosed phenomenon in patients with cancer. The currently available evidence shows that well-designed exercise is effective in alleviating CRF [27]. In breast cancer patients, fatigue was found to be one of the major limiting factors in return to work [28]. The results of previous studies were inconsistent and further investigations with larger sample sizes have been warranted in current literature.

The present study aims to identify the effect of DMT on breast cancer patients with persistent CRF which is a common side effect of RT. The purpose of DMT is to help people to achieve self-awareness and to come in contact with conscious and unconscious parts of one’s own personality during each session. It demonstrated significant improvement in mood after each DMT session. There was a decreased sense of fear, improvement in the overall attitude and feeling of joy which helped them to fight the chronic disease with self-confidence [29].

The results of the present study showed that DMT seems to be effective in reducing fatigue up to a certain level (t= 2.62, p<0.001) which is in accordance with the study conducted by Ho et al., who investigated DMT during RT and demonstrated significant improvement in stress, pain and fatigue. The study stated that there was potential clinical value of DMT in pain reduction and DMT could be incorporated as part of an integrative cancer care as prophylaxis at the start of RT. Although Ho et al. reported a large treatment effect of DMT on pain severity reduction, our DMT group showed no significant effect on improvement of quality of life [30,31]. The lack of significant findings could be attributed to the short duration (2 weeks) of the intervention. Given the time constraint in relation to the RT which is given for 5 weeks, dramatic changes in quality of life or emotional changes were not expected. There was a significant decrease in fatigue after three to four sessions but reoccurred after the gap of 2 days in a week as subjectively reported by the participants. The patients complained of obstinate fatigue after the RT. Hence, the preferable time for the intervention was in the morning hours [32].

The limitations of the study included the recruitment of only ambulatory patients. Thus, the result cannot be generalised to those patients who were less mobile. Patients who were in severe pain and had severe side effects of nausea and vomiting were not included in the program. They lacked motivation or stamina to participate in the program. This may lead to sampling bias to the participants who were not able to participate, although simple random sampling method was used. In general, the elder age group had more pain than the younger [33].

Dance represents a psychotherapeutic treatment and a form of physical activity, based on body awareness, expression and acceptance, in order to facilitate the physical, emotional, cognitive and spiritual integration, thus, making it a holistic approach for cancer care. DMT has shown promising initial result in improving quality of life and mental health of patients with breast cancer [7,34]. It is possible to remark that DMT seems to be helpful in reducing CRF up to a certain level by improving the mental and emotional status of breast cancer patients but further studies are required [21].

## Conclusions

The result of the present study revealed that DMT seems to be effective in reducing some amount of CRF in breast cancer patients undergoing RT. The intervention given for 2 weeks for 5 days per week showed that DMT can be helpful in reducing the side effects of RT such as pain, stress, anxiety and fear, giving a psychotherapeutic relief but did not completely reduce the persistent fatigue experienced by the breast cancer patients. It is engaging, entertaining and cost-effective at the same time. DMT with different dance forms in context of culture can be considered as a complementary therapy for patients with various types of cancer in all age groups. This study opens up avenues to explore physiotherapeutic movements in the management of CRF warranting further investigation with larger sample size and long-term follow-up.

## Figures and Tables

**Figure 2:**
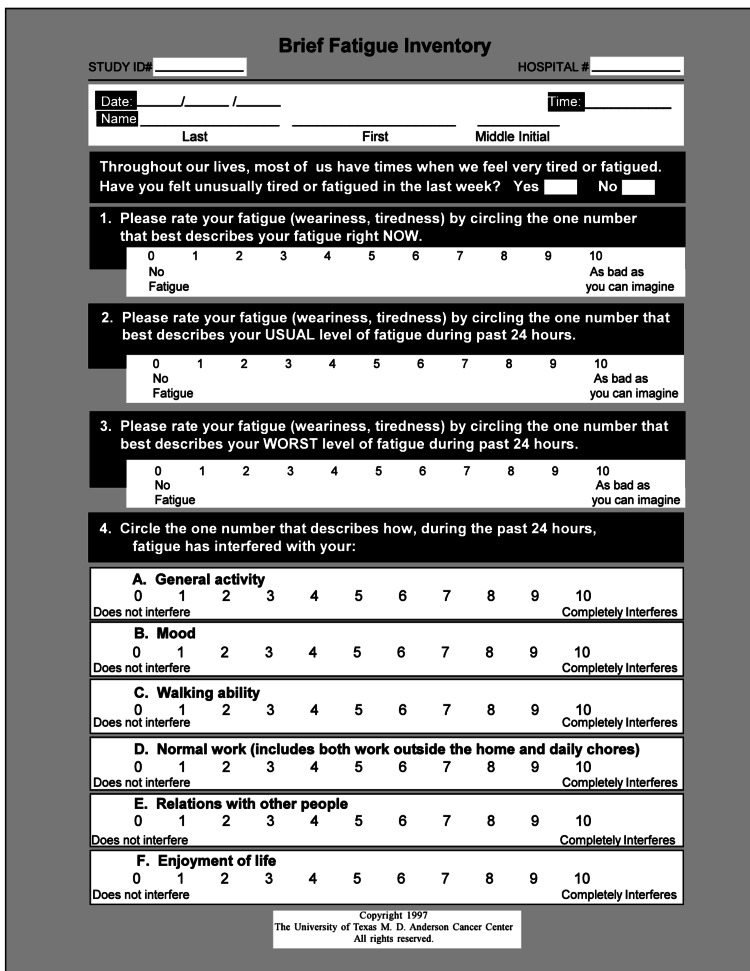
Brief Fatigue Inventory
